# Strengthened second law for multi-dimensional systems coupled to multiple thermodynamic reservoirs

**DOI:** 10.1098/rsta.2020.0428

**Published:** 2022-07-11

**Authors:** David H. Wolpert

**Affiliations:** ^1^ Santa Fe Institute, Santa Fe, NM, USA; ^2^ Complexity Science Hub, Vienna, Arizona State University, Tempe, AZ, USA; ^3^ International Center for Theoretical Physics, Italy

**Keywords:** stochastic thermodynamics, multi-dimensional systems, multipartite processes, entropy production, feedback control, second law of thermodynamics

## Abstract

The second law of thermodynamics can be formulated as a restriction on the evolution of the entropy of any system undergoing Markovian dynamics. Here I show that this form of the second law is strengthened for multi-dimensional, complex systems, coupled to multiple thermodynamic reservoirs, if we have a set of *a priori* constraints restricting how the dynamics of each coordinate can depend on the other coordinates. As an example, this strengthened second law (SSL) applies to complex systems composed of multiple physically separated, co-evolving subsystems, each identified as a coordinate of the overall system. In this example, the constraints concern how the dynamics of some subsystems are allowed to depend on the states of the other subsystems. Importantly, the SSL applies to such complex systems even if some of its subsystems can change state simultaneously, which is prohibited in a multipartite process. The SSL also strengthens previously derived bounds on how much work can be extracted from a system using feedback control, if the system is multi-dimensional. Importantly, the SSL does not require local detailed balance. So it potentially applies to complex systems ranging from interacting economic agents to co-evolving biological species.

This article is part of the theme issue ‘Emergent phenomena in complex physical and socio-technical systems: from cells to societies’.

## 1. Introduction

Statistical physics concerns experimental scenarios where we have restricted information concerning the state of a system x∈X, which is quantified as a probability distribution over those states, $p_{x}(t)$. In particular, the recently developed variant of statistical physics called ‘stochastic thermodynamics’ concentrates on systems that evolve according to a continuous-time Markov chain (CTMC). For a countable state space, this means that $p_{x}(t)$ evolves according to a linear differential equation

1.1 $$\frac{dp_{x}(t)}{dt}=\sum_{x^{\prime }}K_{x}^{x^{\prime }}(t)p_{x^{\prime }}(t).$$

(Note that the rate matrix K(t) can depend on time t.)

Analysing systems that evolve according to equation (1.1) has led to formulations of the second law of thermodynamics which apply even if the system is evolving while arbitrarily far out of thermal equilibrium [1,2]. If we apply one of these formulations of the second law to any system evolving according to equation (1.2) while coupled to a single (infinite) heat bath at temperature T, and assume that the rate matrix is related to an underlying Hamiltonian via local detailed balance (LDB), we get

1.2 $$\frac{Q}{T}\leq \Delta S,$$

where Q is the total heat flow into the system from its heat bath during the dynamics, and ΔS is the change in Shannon entropy of the system during the process.

If LDB does not hold, equation (1.2) will not hold either, if we wish to interpret Q as thermodynamic heat flow. However, for any rate matrix, regardless of whether it obeys LDB,

1.3 $$\int_{t_{i}}^{t_{f}}\;dt\sum_{x^{\prime }}K_{x}^{x^{\prime }}(t)p_{x^{\prime }}(t)\ln\frac{K_{x^{\prime }}^{x}(t)}{K_{x}^{x^{\prime }}(t)}\leq \Delta S$$

(for a process lasting from time $t_{i}$ to $t_{f}$). The quantity on the l.h.s. of equation (1.3) is called the total expected entropy flow (EF) into the system during the process. The difference between the entropy change of the system (the r.h.s. of equation (1.3)) and the EF is called the entropy production (EP), written as σ. So equation (1.3) can be re-expressed as

1.4 σ≥0.

Crucially, the inequality equation (1.4) holds for *any* CTMC, even a CTMC that has no thermodynamic interpretation, i.e. a CTMC which models a process that does not involve energy transduction. So equation (1.4) applies to dynamic models of everything from stock markets to the evolution of the joint state of an opinion network, so long as those models are CTMCs.

In many experimental scenarios, while we are restricted in the information we have concerning the system’s state, we have some other information, in the form of conditions satisfied by the dynamics of the system. Recently, equation (1.4) has been strengthened, by adding non-positive terms to the r.h.s. that incorporate this kind of information concerning the dynamics. Examples of these new results include ‘thermodynamic uncertainty relations’ (TURs [3–6]), ‘speed limit theorems’ (SLTs [7–11]), ‘thermodynamic first passage bounds’ [12–16], etc.

Unlike equation (1.4) though, these bounds require measuring variables as they change during the process, in addition to knowing the beginning and ending distributions, $p_{x}(t_{i})$ and $p_{x}(t_{f})$. (For example, TURs rely on measuring accumulated currents, and SLTs rely on measuring integrated activity.) This limits their experimental applicability.

In this paper, I derive new strengthened forms of equation (1.4) that, like the TURs and SLTs, incorporate information concerning the dynamics of the system. However, unlike the TURs, SLTs, etc., these new strengthened forms of equation (1.4) do *not* require measuring variables as they change during the process; they only require knowing the beginning and ending distributions over states.

These strengthened forms of equation (1.4) apply whenever we have information about which of the coordinates of the system can have their dynamics directly depend on which of the other coordinates. Formally, such information takes the form of constraints on the rate matrix K(t) of the CTMC governing the dynamics of the system. (See also [17].) I call this kind of restriction on the allowed dynamics a ‘dependency constraint’.

As an example, consider a random walker over a two-dimensional finite lattice, $Y_{1}\times Y_{2}$. For simplicity take $|Y_{1}|=|Y_{2}|=LN$ for two positive integers L,N. The lattice is coarse-grained into a set of $N^{2}$ non-overlapping squares each of size L×L, and the position of the walker in the lattice is represented three-dimensionally, by a pair of coordinates $X_{1}=\{1,\ldots ,L\},X_{2}=\{1,\ldots ,L\}$ and an integer $X_{3}\in \{1,\ldots ,N^{2}\}$. (The value $x_{3}\in X_{3}$ specifies the precise coarse-grained square, while $(x_{1},x_{2})\in X_{1}\times X_{2}$ specifies the coordinates within that square.) In addition to position in the lattice, the walker has internal stores of two nutrients, A and B, specified (up to some coarse-graining) by values in the finite sets $X_{A}$ and $X_{B}$, respectively. So the state space of the walker is $X=X_{A}\times X_{B}\times \prod_{i=1}^{3}X_{i}$, i.e. those five variables are the five coordinates of the walker.

We can suppose that both $x_{1}$ and $x_{2}$ evolve autonomously, independently of all other variables, according to two associated rate matrices, i.e. the walker engages in two independent random walks, one in each of the two directions across the lattice. Note though that $x_{3}$’s dynamics will depend on $x_{1}$ and $x_{2}$ in general, and that there will sometimes be simultaneous transitions of $x_{3}$ and some other coordinate. For example, suppose $x_{1}=L$, so the walker is at the extreme value of $X_{1}$ within some square, adjacent to the next coarse-grained square. Suppose as well that in the next step, the walker moves into that adjacent square. So simultaneously $x_{1}$ changes to 1 while $x_{3}$ must also change, since the coarse-grained square changes. However, for other changes in $x_{1}$, $x_{3}$ remains unchanged.

We can also suppose that the dynamics of $x_{A}\in X_{A}$ depends only on the walker’s current position in $X_{1}$ and their current amount of $x_{A}$, i.e. it depends only on $\left(x_{A},x_{1}\right)$. Similarly, the dynamics of $x_{B}\in X_{B}$ depends only on $\left(x_{B},x_{2}\right)$. (For example, this would be the case if densities of those two nutrients were arranged appropriately across the lattice, and the walker at a given location accumulates those nutrients based on their densities at that location.) Summarizing, the dependency constraints are that $x_{A}$ depends only on $x_{1}$ (in addition to depending on its own state), $x_{B}$ depends only on $x_{2}$ (in addition to its own state), $x_{1}$ and $x_{2}$ are autonomous, while $x_{3}$ can depend on $x_{1}$ and/or $x_{2}$ (in addition to itself).

**Figure 1.**
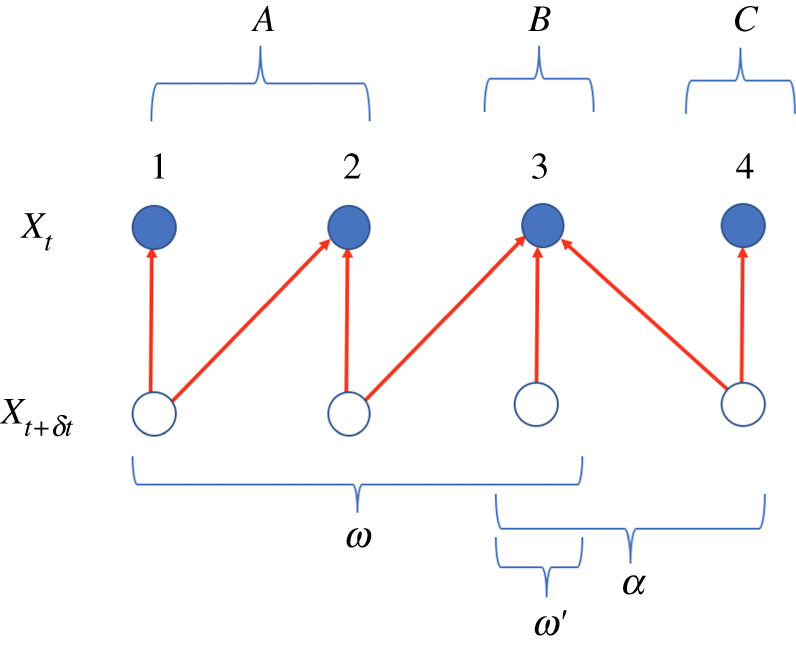
Four interacting subsystems, {1,2,3,4}, grouped into three sets, {A,B,C}. The red arrows indicate dependencies in rate matrix of the overall system. So for example B evolves autonomously, but is continually observed by A and C. (The implicit assumption that B is not affected by the back-action of the observation holds for many real systems such as colloidal particles and macromolecules [23].) Note that the statistical coupling between A and C could grow with time, even though the rate matrix does not directly couple their dynamics. The three overlapping sets indicated at the bottom of the figure specify the three units of a unit structure for this process, as discussed in the text. As an illustration of some of the definitions below, there is one reservoir coupled to the system that has subsystem 2 as its puppet set, with both subsystems 2, 3 as its leader set. (Online version in colour.)

However, for this special issue on the topic of ‘Emergence’, perhaps the most important type of system that evolves subject to dependency constraints is a system that comprises a set of physically separated subsystems, co-evolving with one another, with each subsystem’s state being identified as a different coordinate [18–20]. In this kind of system, dependency constraints governing the dynamics of each coordinate, specifying which other coordinates can directly affect its dynamics, amount to constraints on the dynamics of each subsystem, specifying which other subsystems can directly affect its dynamics. As a concrete illustration, consider the scenario investigated in [21,22], in which receptors in the wall of a cell sense the concentration of a ligand in the intercellular medium, and those receptors are in turn observed by a ‘memory’ subsystem inside the cell. Modify this scenario by introducing a second cell, which is observing the same external medium as the first cell. Assume that the cells are far enough apart physically so that their dynamics are independent of one another. This gives us the precise scenario in figure 1, where subsystem 3 is concentration in the external medium, subsystem 2 is the state of the receptors of the first cell, subsystem 1 is the memory subsystem of the first cell and subsystem 4 is the state of the receptors of the second cell.

My main result shows how a set of dependency constraints can strengthen equation (1.4), by adding an expression to its r.h.s.. This expression involves only those dependency constraints and the starting and ending distribution of the system. As a caveat, this new lower bound on EP is not always positive, i.e. it is not always stronger than the conventional second law, equation (1.4). However, I show below that for any set of dependency constraints, there is a conditional distribution $p(x(t_{f})\;|\;x(t_{i}))$ that can be implemented by a rate matrix obeying those constraints, together with an initial distribution $p(x(t_{i}))$, such that *every* rate matrix that implements that conditional distribution must result in a non-negative EP when applied to that initial distribution. Indeed, for some sets of dependency constraints, this new EP bound is stronger than the conventional second law no matter what $p(x(t_{i}))$ and $p(x(t_{f})\;|\;x(t_{i}))$ are (so long as $p(x(t_{f})\;|\;x(t_{i}))$ is consistent with the dependency constraints).

Some of the TURs, SLTs, etc., rely on the dynamics obeying LDB. LDB is not required for the new extension of the second law derived here. This means that (for example) this new extension applies to multipartite systems that have ‘directed’ (sometimes called ‘non-reciprocal’) interactions rather than undirected interactions among the subsystems, i.e. interactions in which there is exactly zero back-action [22,24–30]. Very often, these systems violate strict LDB, and so their thermodynamic analyses are, at best, approximations. (See discussion in appendix in [19] of some conditions that justify this approximation.) By contrast, the result derived below applies exactly to any scenario where there is no back-action, with no approximation. In addition, this result holds even if the dynamics allows multiple coordinates to change simultaneously. In particular, in the special case that each coordinate is a separate subsystem, the result does not require that the dynamics be a multipartite process (MPP) [18].

Owing to these relaxations of the assumptions made in conventional stochastic thermodynamics, the results below are not restricted to thermodynamic systems, involving energy transduction. The results hold for any CTMC, even if the rate matrix does not reflect physically coupling between the system and one or more external thermodynamic reservoirs, as it does in conventional applications of stochastic thermodynamics [2].

However, the strengthened second law derived below has special physical significance in the common scenario where the dependency constraints arise because the system’s dynamics is governed by coupling with external reservoirs, and there are restrictions on that coupling. For example, a common physical scenario is where the system has multiple subsystems, and each subsystem is coupled to a physically distinct part of a shared reservoir. Owing to the physical separation of those parts of the reservoir, each connected to a different subsystem, the usual assumption of time-scale separation between the dynamics of the overall system and that of the reservoirs means that the different subsystems are effectively coupled to independent reservoirs from one another.^1^ Such systems evolve as an MPP, in which no transitions are allowed in which more than two subsystems change their states exactly simultaneously [18]. If the system is an MPP, and the dynamics of each subsystem obeys LDB, then we can use stochastic thermodynamics to identify various attributes of that dynamics with experimentally measurable thermodynamic quantities [18,24,33–35]. More generally, there are systems with multiple coordinates that are not usually viewed as separate ‘subsystems’, but where the global dynamics arises due to the system’s coupling with thermodynamic reservoirs, and where each reservoir is only coupled to a single coordinate. These systems can also be modelled as MPPs, and analysed accordingly.

Generalizing further, there are other kinds of systems that also have multiple coordinates, where the global dynamics arises due to the system’s coupling with thermodynamic reservoirs, just like in an MPP. Also like in an MPP, each reservoir in these systems is only coupled to a proper subset of the coordinates, which results in dependency constraints. In contrast to an MPP however, some reservoirs are coupled to more than one coordinate. As an example, as stated in [36]: ‘Fluctuations in biochemical networks, e.g. in a living cell, have a complex origin that precludes a description of such systems in terms of bipartite or MPPs, as is usually done in the framework of stochastic and/or information thermodynamics’. The strengthened second law I present below applies to these generalized forms of MPPs as well as to MPPs.

In the next section, I formalize dependency constraints as restrictions on the rate matrix of a CTMC. This is followed by a section in which I use this formalization to derive an expression for the EP of a system that involves the triple of {the rate matrix dependency constraints, the initial distribution over states, the final distribution over states}, together with certain other factors. In the following section I derive a lower bound on that expression for EP which depends only on the triple of {dependency constraints, initial distribution, final distribution}, without those other factors. In particular, this lower bound does not depend on any properties of the rate matrix, other than the dependency constraints. This lower bound is my main result. In the following section, this main result to analyse how the thermodynamics of feedback control [17,27,28] changes when we know that the system being controlled obeys a given set of dependency constraints. In the following section, I present a set of examples of my main result. I end with some discussion, in particular of the relation of the new result to other results in the literature.

## 2. Rate matrix unit structures

I begin by defining notation. First, I write the state space of the system as $X=\prod_{i=1}^{N}X_{i}$, where each finite state space $X_{i}$ is a coordinate of the system. I write the set of N coordinates as N. As examples, each coordinate could specify the state of a physically separate subsystem of the overall system, or it could specify a position on one axis of a lattice, or it could indicate a degree of freedom in a multiscale specification of the state of the system.

The system is assumed to evolve according to a CTMC.^2^ For any A⊂N, I write −A:=N∖A. So for example, $x_{-A}$ is the vector of all components of x other than those in A. For any set L, $\Delta_{L}$ is the associated unit-simplex. In addition, for any function f(p), I write $\Delta f:=f(p_{t_{f}})-f(p_{t_{i}})$. The set of bits is B={0,1}. I write the Kronecker delta as δ(a,b). For any family of sets, $A=\{a_{1},a_{2},\ldots \}$, I define $\cup A=a_{1}\cup a_{2}\cup \ldots$.

A distribution over a set of values x at time t is written as $p_{X}(t)$, with its value for x∈X written as p(x(t)). Similarly, I write $p(x(t)\;|\;x(t^{\prime }))$, for the conditional distribution of the state at time t given the state at time $t^{\prime }$, etc. I write Shannon entropy as $S(p_{X}(t))$, $S_{t}(X)$, or $S^{X}(t)$, depending on which would result in the cleanest equations, and write mutual information between two random variables F,G as I(F;G).

The distribution over the overall system evolves according to the global rate matrix K(t), as given by equation (1.1). A unit ω⊆N at time t is a set of coordinates such that as the full system evolves according to K(t), the marginal distribution $p_{x_{\omega }}$ evolves according to the CTMC

2.1 $$\frac{dp_{x_{\omega }}(t)}{dt}=\sum_{x_{\omega }^{\prime }}K_{x_{\omega }}^{x_{\omega }^{\prime }}(\omega ;t)p_{x_{\omega }^{\prime }}(t),$$

for all p, for some associated rate matrix K(ω;t). Intuitively, a unit is any set of coordinates whose evolution is independent of the states of the coordinates outside the unit. Since the dynamics of a unit is given by a self-contained CTMC, all the usual theorems of stochastic thermodynamics apply to any unit, e.g. the second law [2], SLTs [9,37], some of the fluctuation theorems [38] and if LDB holds, then other fluctuation theorems [1], the thermodynamic uncertainty relations [4,6,39], first-passage time bounds [13], bounds on stopping times [15], etc.

Any union of units is a unit. In addition, it is proven in electronic supplementary material, appendix A that any non-empty intersection of units is a unit. Note that since the dynamics of the full system is a CTMC, equation (2.1) applies with ω set to all coordinates in the system. So N is a unit. Note also that, in general, the evolution of a coordinate i lying outside of a unit ω may depend on the states of coordinates j lying inside ω, even though the reverse is impossible by definition.

As an example, in figure 1, subsystem 3 is its own unit, evolving independently of subsystems 2 and 1. By contrast, none of the other three subsystems are their own unit. (For example subsystem 2’s dynamics depends on the state of 3.)

A set of units defined over a set of coordinates N is called a unit structure if it obeys the following properties [19,20]:

(i) The union of the units in the unit structure equals all of N.
(ii) The unit structure is closed under intersections of its units.

I will generically write any particular unit structure defined over N as $N^{*}$.^3^

I will sometimes say that $N^{*}$ represents the set of coordinates N. Any process can be represented with at least one unit structure, e.g. by choosing a unit structure that contains only the single unit N. (Typically, it can be represented by many possible unit structures.) Also, in general for any given rate matrix there are sets of coordinates A⊂N that are not unions of units, and so cannot be represented by any unit structure. On the other hand, one can always construct a rate matrix that will implement any hypothesized unit structure over a set of coordinates, i.e. all unit structures can actually exist, for some appropriate rate matrix. (At worst, one can choose a rate matrix in which each coordinate evolves autonomously, i.e. a rate matrix that is a sum over all coordinates of independent rate matrices for each of those coordinates.) For simplicity, from now on I assume that the unit structure does not change with t. In addition, I define a conditional distribution for the ending joint state given an initial joint state, $p(x(t_{f})\;|\;x(t_{i}))$, to be consistent with a specified unit structure if there is some rate matrix that obeys that unit structure and that implements $p(x(t_{f})\;|\;x(t_{i}))$.

The dynamics of any two units ω,α⊂ω must be consistent with one another, i.e. for all $p_{x_{\omega }}(t)=p_{x_{\alpha },x_{\omega \setminus \alpha }}(t)$,

2.2 $$\sum_{x_{\alpha }^{\prime }}K_{x_{\alpha }}^{x_{\alpha }^{\prime }}(\alpha ;t)p_{x_{\alpha }^{\prime }}(t)=\sum_{x_{\omega \setminus \alpha }^{\prime }}\sum_{x_{\omega \setminus \alpha }}K_{x_{\alpha },x_{\omega \setminus \alpha }}^{x_{\alpha }^{\prime },x_{\omega \setminus \alpha }^{\prime }}(\omega ;t)p_{x_{\alpha }^{\prime },x_{\omega \setminus \alpha }^{\prime }}(t)$$

i.e.,

2.3 $$\sum_{x_{\alpha }^{\prime }}K_{x_{\alpha }}^{x_{\alpha }^{\prime }}(\alpha ;t)\sum_{x_{\omega \setminus \alpha }^{\prime }}p_{x_{\alpha }^{\prime },x_{\omega \setminus \alpha }^{\prime }}(t)=\sum_{x_{\omega \setminus \alpha }}\sum_{x_{\alpha }^{\prime }}\sum_{x_{\omega \setminus \alpha }^{\prime }}K_{x_{\alpha },x_{\omega \setminus \alpha }}^{x_{\alpha }^{\prime },x_{\omega \setminus \alpha }^{\prime }}(\omega ;t)p_{x_{\alpha }^{\prime },x_{\omega \setminus \alpha }^{\prime }}(t)$$

(Note that the l.h.s. can be expanded as $\sum_{x_{\alpha }^{\prime }}K_{x_{\alpha }}^{x_{\alpha }^{\prime }}(\alpha ;t)\sum_{x_{\omega \setminus \alpha }^{\prime }}p_{x_{\alpha }^{\prime },x_{\omega \setminus \alpha }^{\prime }}(t)$.) In particular equation (2.2) must hold for

2.4 $$p_{x_{\alpha }^{\prime },x_{\omega \setminus \alpha }^{\prime }}(t)=\delta ([x_{\alpha }^{\prime },x_{\omega \setminus \alpha }^{\prime }],[x_{\alpha }^{\prime \prime },x_{\omega \setminus \alpha }^{\prime \prime }]),$$

for any joint state $\left[x_{\alpha }^{\prime \prime },x_{\omega \setminus \alpha }^{\prime \prime }\right]$. If we use equation (2.1) to evaluate the derivative on the l.h.s. and r.h.s. of equation (2.2) and apply it for all such delta function choices of $p_{x_{\alpha }^{\prime },x_{\omega \setminus \alpha }^{\prime }}(t)$ and then relabel, we see that for all $x_{\alpha },x_{\alpha }^{\prime }$ and $x_{\omega \setminus \alpha }^{\prime }$,

2.5 $$K_{x_{\alpha }}^{x_{\alpha }^{\prime }}(\alpha ;t)=\sum_{x_{\omega \setminus \alpha }}K_{x_{\alpha },x_{\omega \setminus \alpha }}^{x_{\alpha }^{\prime },x_{\omega \setminus \alpha }^{\prime }}(\omega ;t)$$

(See electronic supplementary material, appendix B for more discussion of this result.) Conversely, if there is some rate matrix $K_{x_{\alpha }}^{x_{\alpha }^{\prime }}(\alpha ;t)$ such that equation (2.5) holds for all $x_{\omega \setminus \alpha }^{\prime }$, then the two rate matrices are compatible, i.e. equation (2.2) holds. As an important special case of equation (2.5), if we take ω=N and as shorthand writing K(N;t) as just K(t), we see that for any unit α,

2.6 $$K_{x_{\alpha }}^{x_{\alpha }^{\prime }}(\alpha ;t)=\sum_{x_{-\alpha }}K_{x_{\alpha },x_{-\alpha }}^{x_{\alpha }^{\prime },x_{-\alpha }^{\prime }}(t),$$

independent of $x_{-\alpha }^{\prime }$.

**Example 2.1.**

Recall that an MPP is a set of co-evolving subsystems evolving according to a CTMC in which no transitions are allowed in which more than two subsystems both change their states. Formally, in an MPP, for all subsystems i, for all $x^{\prime }$, x, $K_{x_{i},x_{-i}}^{x_{i}^{\prime },x_{-i}^{\prime }}(t)=0$ unless $x_{-i}^{\prime }=x_{-i}$ [17,18]. The units in an MPP are sets of subsystems whose joint evolution is independent of the other subsystems. Equivalently, for every subsystem i in an MPP, there is an associated rate matrix $K_{x}^{x^{\prime }}(i;t)$ that is zero if $x_{-i}^{\prime }\neq x_{-i}$ such that the global rate K matrix can be written as

2.7 $$K_{x}^{x^{\prime }}(t)=\sum_{i\in N}K_{x_{i},x_{-i}}^{x^{\prime },x_{-i}^{\prime }}(i;t)$$

and where for every unit ω containing subsystem i, the rate matrix terms $K_{x_{\omega },x_{-\omega }^{\prime }}^{x_{\omega }^{\prime },x_{-\omega }^{\prime }}(i;t)$ are independent of $x_{-\omega }^{\prime }$.

Equation (2.2) always holds (and therefore so does equation (2.5)) in an MPP. At the other extreme from MPPs, equation (2.5) also holds for some rate matrices K(t) which only allow state transitions in which *all* subsystems change, i.e. rate matrices K(t) such that $K_{x}^{x^{\prime }}(t)=0$ for any $x,x^{\prime }$ where there are two subsystems, j, k such that both $x_{j}\neq x_{j}^{\prime }$ and $x_{k}=x_{k}^{\prime }$. This is illustrated in electronic supplementary material, appendix B.

It will often be convenient to re-express a unit structure as a directed graph. Define the dependency graph $\Gamma_{N^{*}}=(N^{*},E)$ by the rule that there is an edge e∈E from node $\omega \in N^{*}$ to node $\omega^{\prime }\in N^{*}$ iff both: $\omega^{\prime }\subseteq \omega$, and there is no intervening unit $\omega^{\prime \prime }$ such that $\omega^{\prime }\subseteq \omega^{\prime \prime }\subseteq \omega$. (Note that $\Gamma_{N^{*}}$ is a directed graph, which allows us to use standard graph theory terminology.) In a unit structure $N^{*}$ where $N\in N^{*}$ the dependency graph has a single root, but if $N\notin N^{*}$, then the dependency graph has multiple roots.

I will abuse notation and sometimes treat a unit ω as a set of coordinates while at other times I treat it as a single node in $\Gamma_{N^{*}}$. I write the set of parents of any node $\omega \in \Gamma_{N^{*}}$ as pa(ω), and the set of its descendants as desc(ω), with fa(ω):=ω∪desc(ω), the family of node ω. The maximal number of nodes in any directed path that starts at ω is the height of ω. So any unit ω that has no subunits contained in it is a leaf node of $\Gamma_{N^{*}}$, with height 1. (The maximal height of all nodes in $\Gamma_{N^{*}}$ is simply called ‘the height of $N^{*}$’.) I write $\Gamma_{N^{*}}^{R}$ for the set of root nodes in $\Gamma_{N^{*}}$. As an example, the dependency graph of figure 1 has two root nodes, ω and α, and one leaf node, $\omega^{\prime }$, which is their common child. The height of the graph is 2.

For simplicity, from now on I assume that neither the number of reservoirs nor the associated maps P(.) and L(.) changes with time t. There are several additional, technical conditions that I will impose on the unit structure, in order to simplify the algebra in the proofs of the results in §4. (These conditions can be ignored if the reader is only interested in understanding the results, not the details of their proofs.)

(i) I require that the unit structure is rich enough that if a joint state transition can occur that simultaneously changes the state of all coordinates in a set α, then there is some unit $\omega \in N^{*}$ that contains α.^4^ I call such a unit structure flush.
(i) A unit ω is vacuous if all of its coordinates are also in at least one subunit $\omega^{\prime }\subseteq \omega$. I assume that no unit in any unit structure we are considering is vacuous.^5^
(iii) I say that two units $\omega ,\omega^{\prime }\subset \omega$ are equivalent at time t if for all $x^{\prime }$ where $p_{x^{\prime }}(t)\neq 0$, for all x such that $x_{\omega \setminus \omega^{\prime }}^{\prime }\neq x_{\omega \setminus \omega^{\prime }}$, $K_{x}^{x^{\prime }}(\omega ;t)=0$. I require that $N^{*}$ does not contain any two equivalent units. This means that for any two units $\omega ,\omega^{\prime }\subset \omega$ in the unit structure, there must be transitions $x^{\prime }\to x$ that can occur in which some coordinate $i\in \omega \setminus \omega^{\prime }$ changes its value.

Any CTMC can be represented with at least one unit structure meeting these three conditions (e.g. the unit structure that consists just of {N}).

To connect these considerations to stochastic thermodynamics, from now on I suppose there are a total of R thermodynamic reservoirs attached to the system [1,2]. I suppose further that each reservoir v∈{1,…,R} generates fluctuations of the joint state of an associated set of coordinates P(v)⊆N, without any such direct effect on the other coordinates. (For example, v may be able to do this by being directly physically coupled to the coordinates in P(v) and no others, via an implicit interaction Hamiltonian.) As is standard in stochastic thermodynamics, I suppose that if only one particular reservoir v were attached to the system, then the resultant dynamics over P(v) would be a CTMC. P(v) is called the puppet set of reservoir v, with its elements called the puppets of v. The collection of all R puppet sets covers N. To minimize the amount of notation required, I assume that the set-valued function P(.) is invertible, i.e. a given pair of reservoirs v, $v^{\prime }\neq v$ might both affect the dynamics of some shared coordinate i, but there will always be at least one coordinate j whose dynamics is not affected by both those reservoirs v and $v^{\prime }$.

**Example 2.2.**

Return to the example of an MPP, where we identify each subsystem with a separate coordinate. Each subsystem has its own unique set of reservoirs, which jointly causes the fluctuations in its state. In other words, the puppet set of each reservoir is a singleton, the associated subsystem of that reservoir, and each subsystem is the puppet set of at least one reservoir.

I write L(v)⊇P(v) for the minimal set of coordinates whose associated value directly affects how the coupling with reservoir v affects the dynamics of $x_{P(v)}$. I call this the leader set of P(v), or sometimes the leader set of v.^6^ I write L(v;t) for the associated rate matrix over X induced by the coupling of the system to reservoir v. So L(v;t) affects the dynamics of $x_{P(v)}$, but leaves the other coordinates unchanged. Abusing notation, I write

2.8 $$\begin{array}{rl} L_{x}^{x^{\prime }}(v;t) & \;=L_{x_{P(v)},x_{L(v)\setminus P(v)}}^{x_{L(v)}^{\prime }}(v;t)\delta_{x_{-P(v)}}^{x_{-P(v)}^{\prime }} \end{array}$$

where $L_{x_{P(v)},x_{L(v)\setminus P(v)}}^{x_{L(v)}^{\prime }}(v;t)$ is a proper stochastic rate matrix that equals 0 if $x_{L(v)\setminus P(v)}\neq x_{L(v)\setminus P(v)}^{\prime }$. Abusing notation, I will sometimes rewrite equation (2.8) as

2.9 $$\begin{array}{rl} L_{x}^{x^{\prime }}(v;t) & \;=L_{x_{P(v)}}^{x_{L(v)}^{\prime }}(v;t)\delta_{x_{-P(v)}}^{x_{-P(v)}^{\prime }} \end{array}$$

where $L_{x_{P(v)}}^{x_{L(v)}^{\prime }}(v;t)$ is a ‘rate matrix’ in that all of its entries for $x_{P(v)}^{\prime }\neq x_{P(v)}$ are non-negative, and

2.10 $$\begin{array}{r} \sum_{x_{P(v)}}L_{x_{P(v)}}^{x_{L(v)}^{\prime }}(v;t)=0 \end{array}$$

See figure 1 above and example 3.1 below.

In general, any given coordinate i may be in more than one reservoir's leader set and in more than one reservoir's puppet set. Accordingly, I extend the definitions above by writing

2.11 $$\begin{array}{rl} L(i) & \;:=\underset{v:i\in P(v)}{⋃}L(v)\\ L(A) & \;:=\underset{i\in A}{⋃}L(i) \end{array}$$

where A is an any subset of N. So L(i) is the set of all coordinates whose state can directly affect the dynamics of coordinate i, via arguments of a rate matrix, and similarly for L(A). Along the same lines, I define

2.13 $$\begin{array}{rl} P(i) & \;:=\underset{v:i\in P(v)}{⋃}P(v)\\ P(A) & \;:=\underset{i\in A}{⋃}P(i) \end{array}$$

So P(A) is the set of all coordinates, inside or outside of A, whose dynamics is governed jointly with that of any coordinate in A. L(v)⊆L(P(v)), since there can be coordinates i∈P(v) whose dynamics is affected by other reservoirs in addition to v. Note as well that for any set A, A⊆P(A)⊆L(A). So in particular, if any two different units have non-empty intersection, then since that intersection must also be a unit, the leader sets of all the coordinates in that intersection must lie within that intersection. In addition, the inverses of these set-valued functions are well-defined. In particular, for any set of coordinates A, $P^{-1}(A)$ is the set of all reservoirs v such that i∈P(v) for some i∈A.

It will be convenient to introduce the shorthand that for any subset A⊆N,ν(A) is the set of all reservoirs v such that P(v)∩A≠∅. So ν(A) is the set of all reservoirs who affect the dynamics of any of the coordinates in A.

As in conventional stochastic thermodynamics, the global rate matrix at time *t* is the sum over all reservoirs of the rate matrices of those reservoirs,

2.15 $$\begin{array}{r} K_{x}^{x^{\prime }}(t)=\sum_{v}L_{x_{P(v)}}^{x_{L(v)}^{\prime }}(v;t)\delta_{x_{{\;}_{-P(v)}}}^{x_{{\;}_{-P(v)}}^{\prime }} \end{array}$$

In appendix K it is shown that this implies the following intuitive result:

**Proposition 2.3.**

*For any unit* ω,

$$K_{x_{\omega }}^{x_{\omega }^{\prime }}(t)=\sum_{v\in \nu (\omega )}{\hat{L}}_{x_{P(v)\cap \omega }}^{x_{L(v)\cap \omega }^{\prime }}(v,\omega ;t)\delta_{x_{{\;}_{\omega \setminus P(v)}}}^{x_{{\;}_{\omega \setminus P(v)}}^{\prime }}$$

*where* ${\hat{L}}_{x_{P(v)\cap \omega }}^{x_{L(v)\cap \omega }^{\prime }}(v,\omega ;t)$ *is a properly normalized rate matrix over* $x_{P(v)\cap \omega }$ *and is independent of* $x_{{\;}_{\omega \setminus P(v)}}^{\prime }$.

**Example 2.4.**

As a simple illustration of Proposition 2.3, in any MPP where each subsystem is controlled by one reservoir, which controls no other subsystems. To reduce notation, consider the case where the unit ω is all of N. In this case the sum over v∈ν(ω) runs over all subsystems i in unit ω, and each ${\hat{L}}_{x_{P(v)\cap \omega }}^{x_{L(v)\cap \omega }^{\prime }}(v,\omega ;t)$ is the rate matrix of the subsystem i associated with reservoir v. So Proposition 2.3 reduces to equation (2.7) in Example 2.1, with each term ${\hat{L}}_{x_{P(v)\cap \omega }}^{x_{L(v)\cap \omega }^{\prime }}(v,\omega ;t)\delta_{x_{{\;}_{\omega \setminus P(v)}}}^{x_{{\;}_{\omega \setminus P(v)}}^{\prime }}$ in Proposition 2.3 re-expressed as $K_{x_{i},x_{-i}}^{x_{i}^{\prime },x_{-i}^{\prime }}(i;t)$.

Proposition 2.3 means that as far as any single unit ω is concerned, we can replace all reservoirs v∈ν(ω) with leader set L(v) and puppet set P(v) with a reservoir that has leader set L(v)∩ω⊆ω and puppet set P(v)∩ω⊆ω. Here I assume that the unit structure has this property simultaneously for all units. Formally, I restrict attention to unit structures that only contains units ω with the property that for all reservoirs v∈ν(ω), L(v)⊆ω. I call such a unit structure **tight**. (Note that there is always at least one unit structure with this property, namely the unit structure with a single element, the unit N.)

For any unit ω in a tight unit structure, L(ω)=ω.^7^ Since A⊆P(A)⊆L(A) for all sets A, it then follows that P(ω)=ω for any unit ω in a tight unit structure. In addition, P(−ω)=−ω in a tight unit structure, even though −ω=N∖ω is not a unit in general.^8^ If LDB holds for all reservoirs v with puppet set inside a unit, then other fluctuation theorems [35], the thermodynamic uncertainty relations [15,18,23], first-passage time bounds [10], bounds on stopping times [27], etc., all apply to the thermodynamics of that unit. (See also [49].)

We can tighten Proposition 2.3 under our assumption of a tight unit structure. The following result is proven in appendix L:

**Proposition 2.5.**

*For any unit* ω *in a tight unit structure*,

$$K_{x_{\omega }}^{x_{\omega }^{\prime }}(\omega ;t)=\sum_{v\in \nu (\omega )}L_{x_{\omega }}^{x_{\omega }^{\prime }}(v;t)$$

## 3. Thermodynamics of composite systems

Following conventional stochastic thermodynamics, I identify the (expected) global EF rate at time t as

3.1 $$\begin{array}{rl} \left\langle \dot{Q}(t)\right\rangle & \;=\sum_{v,x^{\prime },x}L_{x}^{x^{\prime }}(v;t)p_{x^{\prime }}(t)\ln\frac{L_{x}^{x^{\prime }}(v;t)}{L_{x^{\prime }}^{x}(v;t)} \end{array}$$

3.2 $$\begin{array}{rl} & \;=\sum_{v}\sum_{x_{L(v)}^{\prime },\;x_{P(v)}}L_{x_{P(v)}}^{x_{L(v)}^{\prime }}(v;t)p_{x_{L(v)}^{\prime }}(t)\ln\frac{L_{x_{P(v)}}^{x_{L(v)}^{\prime }}(v;t)}{L_{x_{P(v)}^{\prime }}^{x_{L(v)}}(v;t)}. \end{array}$$

The results below do not require LDB. However, if all reservoirs are purely thermal, with no associated particle exchange, and if LDB applies, then we can interpret the EF rate as (temperature-normalized) heat flow between the system and its reservoirs.^9^

Similarly, the (expected) global EP rate at time t is

3.3 $$\begin{array}{rl} \left\langle \dot{\sigma }(t)\right\rangle & \;=\sum_{v,x^{\prime },x}L_{x}^{x^{\prime }}(v;t)p_{x^{\prime }}(t)\ln\frac{L_{x^{\prime }}^{x}(v;t)p_{x}(t)}{L_{x}^{x^{\prime }}(v;t)p_{x^{\prime }}(t)} \end{array}$$

3.4 $$\begin{array}{rl} & \;=\sum_{v}\sum_{x_{L(v)}^{\prime },\;x_{P(v)}}L_{x_{P(v)}}^{x_{L(v)}^{\prime }}(v;t)p_{x_{L(v)}^{\prime }}(t)\ln\frac{L_{x_{P(v)}}^{x_{L(v)}^{\prime }}(v;t)p_{x_{L(v)}^{\prime }}(t)}{L_{x_{P(v)}^{\prime }}^{x_{L(v)}}(v;t)p_{x_{L(v)}^{\prime }}(t)}. \end{array}$$

**Example 3.1.**

In an MPP, each coordinate i is a ‘subsystem’; R=N; there is a bijection between the set of reservoirs and the set of subsystems; and for every reservoir/subsystem i, P(i)={i}. So a unit ω is any set of subsystems such that for all i∈ω, L(i)⊆ω. In addition, equation (3.4) reduces to

3.5 $$\langle \dot{\sigma }(t)\rangle =\sum_{i}\sum_{x_{L(v)}^{\prime },\;x_{i}}L_{x_{i}}^{x_{L(v)}^{\prime }}(v;t)p_{x_{L(v)}^{\prime }}(t)\ln\frac{L_{x_{i}}^{x_{L(v)}^{\prime }}(v;t)p_{x_{L(v)}^{\prime }}(t)}{L_{x_{i}^{\prime }}^{x_{L(v)}}(v;t)p_{x_{L(v)}}(t)}.$$

See [18–20,22,34,40] and figure 1.

Following the same convention as for global EF rate, I define the (expected) local EF rate of any unit ω⊆N at time t as the entropy flow rate into the associated reservoirs:

3.6 $$\begin{array}{rl} \left\langle {\dot{Q}}^{\omega }(t)\right\rangle & \;=\sum_{v\in \nu (\omega )}\sum_{x^{\prime },x}L_{x}^{x^{\prime }}(v;t)p_{x^{\prime }}(t)\ln\frac{L_{x}^{x^{\prime }}(v;t)}{L_{x^{\prime }}^{x}(v;t)} \end{array}$$

3.7 $$\begin{array}{rl} & \;=\sum_{x_{\omega }^{\prime },x_{\omega },v\in \nu (\omega )}L_{x_{\omega }}^{x_{\omega }^{\prime }}(v;t)p_{x_{\omega }^{\prime }}(t)\ln\frac{L_{x_{\omega }}^{x_{\omega }^{\prime }}(v;t)}{L_{x_{\omega }^{\prime }}^{x_{\omega }}(v;t)}. \end{array}$$

Since no reservoir’s puppet set can include both coordinates inside a unit ω and coordinates outside of ω, for any two units $\omega ,\omega^{\prime }$ where $\omega \cap \omega^{\prime }=\emptyset$,

3.8 $$\begin{array}{rl} & \;\langle {\dot{Q}}^{\omega \cup \omega^{\prime }}(t)\rangle =\sum_{x_{\omega }^{\prime },x_{\omega },v\in \nu (\omega \cup \omega^{\prime })}L_{x_{\omega }}^{x_{\omega }^{\prime }}(v;t)p_{x_{\omega }^{\prime }}(t)\ln\frac{L_{x_{\omega }}^{x_{\omega }^{\prime }}(v;t)}{L_{x_{\omega }^{\prime }}^{x_{\omega }}(v;t)} \end{array}$$

3.9 $$\begin{array}{rl} & \;=\sum_{x_{\omega }^{\prime },x_{\omega }}\left[\sum_{v\in \nu (\omega )}L_{x_{\omega }}^{x_{\omega }^{\prime }}(v;t)p_{x_{\omega }^{\prime }}(t)\ln\frac{L_{x_{\omega }}^{x_{\omega }^{\prime }}(v;t)}{L_{x_{\omega }^{\prime }}^{x_{\omega }}(v;t)}+\sum_{v\in \nu (\omega^{\prime })}L_{x_{\omega }}^{x_{\omega }^{\prime }}(v;t)p_{x_{\omega }^{\prime }}(t)\ln\frac{L_{x_{\omega }}^{x_{\omega }^{\prime }}(v;t)}{L_{x_{\omega }^{\prime }}^{x_{\omega }}(v;t)}\right] \end{array}$$

3.10 $$\begin{array}{rl} & \;=\langle {\dot{Q}}^{\omega }(t)\rangle +\langle {\dot{Q}}^{\omega^{\prime }}(t)\rangle . \end{array}$$

So viewed as a function from the set of all units to reals, $\left\langle {\dot{Q}}^{\omega }(t)\right\rangle$ obeys the countable additivity axiom of a signed measure over $S(N^{*})$, the sigma algebra generated by the units in $N^{*}$. This allows us to extend the definition of local EF rate to the sigma algebra $S(N^{*})$ by using the set of values $\left\{\langle {\dot{Q}}^{\omega }(t)\rangle :\omega \in N^{*}\right\}$ to generate an entire signed measure. So for example, for every pair of units $\omega ,\omega^{\prime }\subset \omega$ in $N^{*}$, even if $\omega \setminus \omega^{\prime }\notin N^{*}$,

3.11 $$\langle {\dot{Q}}^{\omega \setminus \omega^{\prime }}(t)\rangle :=\langle {\dot{Q}}^{\omega }(t)\rangle -\langle {\dot{Q}}^{\omega^{\prime }}(t)\rangle .$$

Recall that the dynamics of any unit is given by a self-contained CTMC, independent of the state of any coordinate outside of that unit. Accordingly, the EP rate of a unit is the sum of the derivative of the entropy of the distribution of the joint state of that unit and the EF rate into the reservoirs of that unit. Using Proposition 2.5 to evaluate that entropy derivative and equation (3.7) to evaluate the EF rate, we can evaluate the (expected) local EP rate of ω at time t as

3.12 $$\begin{array}{rl} \left\langle {\dot{\sigma }}^{\omega }(t)\right\rangle & \;=\frac{dS^{\omega }(t)}{dt}+\langle {\dot{Q}}^{\omega }(t)\rangle \end{array}$$

3.13 $$\begin{array}{rl} & \;=\sum_{x_{\omega }^{\prime },x_{\omega },v\in \nu (\omega )}L_{x_{\omega }}^{x_{\omega }^{\prime }}(v;t)p_{x_{\omega }^{\prime }}(t)\ln\left[\frac{L_{x_{\omega }}^{x_{\omega }^{\prime }}(v;t)p_{x_{\omega }^{\prime }}(t)}{L_{x_{\omega }^{\prime }}^{x_{\omega }}(v;t)p_{x_{\omega }}(t)}\right]. \end{array}$$

Accordingly I sometimes write the global EP rate given in equation (3.4) as $\left\langle {\dot{\sigma }}^{N}(t)\right\rangle$. For any unit ω, $\left\langle {\dot{\sigma }}^{\omega }(t)\right\rangle \geq 0$, since $\left\langle {\dot{\sigma }}^{\omega }(t)\right\rangle$ has the usual form of an EP rate of a single system. (See [17] for a discussion of the relation between local EP rates and similar quantities discussed in [18,35,41].)

Write the local EP generated by a unit ω during the process as

3.14 $$\sigma^{\omega }:=\int_{t_{i}}^{t_{f}}\;dt\;\langle {\dot{\sigma }}^{\omega }\rangle ,$$

and similarly write $\sigma^{N}$ for the global EP. (To minimize notation, I adopt the convention that angle brackets are implicit for time-extended thermodynamic quantities, as opposed to rates.) In electronic supplementary material, appendix C, equation (2.4) and the log sum inequality [42] are used to prove that for any two units ω,α⊂ω, not necessarily part of a unit structure, $\left\langle {\dot{\sigma }}^{\omega }(t)\right\rangle \geq \langle {\dot{\sigma }}^{\alpha }(t)\rangle$ at all times t. Therefore

3.15 $$\sigma^{\omega }\geq \sigma^{\alpha }.$$

In particular it is shown in [17,43] that in the special case where there is a set of units $\left\{\alpha_{j}\right\}$ who have no overlap with another, for any unit $\omega ⊃\cup_{j}\alpha_{j}$,

3.16 $$\sigma^{\omega }\geq \sum_{j}\sigma^{\alpha_{j}}.$$

(See also equation (6.4).)

Let $N^{*}=\{\omega_{j}:j=1,2,\ldots ,n\}$ be a unit structure. For simplicity, from now on I assume that $N\notin N^{*}$. Suppose we have a set of real numbers, f, which are indexed by the units $N^{*}$. It will be convenient to use the associated shorthand,

3.17 $${\hat{\sum }}_{\omega \in N^{*}}\;f^{\omega }:=\sum_{j=1}^{n}f^{\omega_{j}}-\sum_{1\leq j<j^{\prime }\leq n}f^{\omega_{j}\cap \omega_{j^{\prime }}}+\sum_{1\leq j<j^{\prime }<j^{\prime \prime }\leq n}f^{\omega_{j}\cap \omega_{j^{\prime }}\cap \omega_{j^{\prime \prime }}}-\ldots$$

(Note that the precise assignment of integer indices to the units in $N^{*}$ is irrelevant.) This quantity is called the inclusion–exclusion sum (or just ‘in-ex sum’ for short) of f for the unit structure $N^{*}$.

Next, define the time-t in-ex information as

3.18 $$I^{N^{*}}:=\left[\hat{\sum_{\omega \in N^{*}}}S^{\omega }\right]-S^{N}=-S^{N}+\sum_{j=1}^{n}S^{\omega_{j}}-\sum_{1\leq j<j^{\prime }\leq n}S^{\omega_{j}\cap \omega_{j^{\prime }}}+\ldots ,$$

where all the terms in the sums on the r.h.s. are marginal entropies over the (distributions over the coordinates in) the indicated units. As an example, if $N^{*}$ consists of two units, $\omega_{1},\omega_{2}$, with no intersection, then the expected in-ex information at time t is just the mutual information between those units at that time. More generally, if there an arbitrary number of units in $N^{*}$ but none of them overlap, then the expected in-ex information is what is called the ‘multi-information’, or ‘total correlation’, among those units [17,44,45].

In electronic supplementary material, appendix D, Rota’s extension of the inclusion–exclusion principle [46] is used to show that in any composite unit structure

3.19 $$\langle {\dot{Q}}^{N}(t)\rangle ={\hat{\sum }}_{\omega \in N^{*}}\langle {\dot{Q}}^{\omega }(t)\rangle .$$

This implies that the global EP rate is

3.20 $$\left\langle {\dot{\sigma }}^{N}(t)\right\rangle =\frac{dS^{N}(t)}{dt}+\langle {\dot{Q}}^{N}(t)\rangle =-\frac{d}{dt}I^{N^{*}}(t)+{\hat{\sum }}_{\omega \in N^{*}}\langle {\dot{\sigma }}^{\omega }(t)\rangle .$$

This is the first major result of this paper.^10^ Integrating equation (3.20) from the start to the end of a process gives

3.21 $$\sigma^{N}={\hat{\sum }}_{\omega \in N^{*}}\sigma^{\omega }-\Delta I^{N^{*}}.$$

As an example of this result, suppose that we have two physically separated subsystems undergoing an MPP, and that subsystem 2 never changes its state, while subsystem 1 executes a map from $p_{x_{1}}(t_{i})$ to $p_{x_{1}}(t_{f})$, independent of the state of $x_{2}$. Note that if the rate matrix of subsystem 1 depends on the state of subsystem 2, i.e. subsystem 1 observes subsystem 2 as it evolves, then there is only one unit rather than two. Accordingly, equation (3.20) tells us that the global EP rate can depend on this property of whether subsystem 1 observes the state of subsystem 2 as subsystem 1 evolves, even though the conditional distribution of subsystem 1’s final state given its initial state, $p(x_{1}(t_{f})|x_{i}(t_{i}),x_{2}(t_{i}))$, is independent of the state of subsystem 2. In general, this effect of the unit structure on the EP will occur whenever the two subsystems are initially statistically coupled. See electronic supplementary material, appendix E for a discussion.

Equation (3.21) applies to any unit structure. In addition, for any unit structure $M^{*}$ over a set of coordinates M⊂ω, equation (3.15) and the fact that the union of a set of units is itself a unit means that $\sigma^{\omega }-\sigma^{M}\geq 0$. Therefore using equation (3.21) to expand $\sigma^{M}$ gives

3.22 $$\sigma^{\omega }-{\hat{\sum }}_{\omega^{\prime }\in M^{*}}\sigma^{\omega^{\prime }}\geq -\Delta I^{M^{*}}.$$

Equation (3.22) holds even if $M^{*}\subset N^{*}$, and at the other extreme, even if no unit in $M^{*}$ is also in $N^{*}$.

## 4. Strengthened second law for composite systems

In general, to evaluate the in-ex sum of local EPs on the r.h.s. of equation (3.21) requires detailed knowledge of the precise rate matrices during the process. However, following Landauer, the goal in this paper is to derive bounds that are independent of those details, depending only on the starting distribution and the conditional distribution of the final state given the initial state. One might hope that one could achieve this goal simply by setting all local EPs to 0 in equation (3.21), giving

4.1 $$\sigma^{N}\geq -\Delta I^{N^{*}}.$$

Below I will sometimes write the r.h.s. of this equation as $B_{N^{*}}$.

Unfortunately, in general it is impossible to have the local EPs of all units =0 in an arbitrary unit structure, even if one uses a quasi-statically slow process. Indeed, the unit structure itself, independent of any other properties of the rate matrix, may mean that it is impossible to have all local EPs=0.^11^

This might seem to imply that we cannot lower-bound the EP as $B_{N^{*}}$. However, recall that in general there are many different unit structures that all apply to the same CTMC. We are free to choose among those unit structures. And as it turns out, no matter what the CTMC is, we can always choose the unit structure in a way that guarantees that equation (4.1) does in fact hold.

I prove this result in several steps. First, in electronic supplementary material, appendices F and G, I derive a set of lower bounds on EP that always apply, no matter what the unit structure. These lower bounds are summarized in electronic supplementary material, proposition F.1, and are my first main result. These bounds are not in the form of equation (4.1) though; while important in their own right, they do not yet achieve our goal.

On the other hand, in general we can represent any CTMC with a unit structure of height 2. (For example, we can do that by combining all coordinates that are not members of a root node of $\Gamma_{N^{*}}$, into one, overarching unit.) In electronic supplementary material, appendix F, I derive a corollary of electronic supplementary material, proposition F.1, telling us that equation (4.1) holds for any such unit structure of height 2. This is my second main result.^12^

Owing to this result, we can always choose the unit structure $N^{*}$ so that the global EP is bounded by equation (4.1). Unfortunately, as illustrated below, there are some unit structures $N^{*}$ of height 2 where the bound on the r.h.s. of equation (4.1) is negative for an appropriate initial distribution $p_{t_{i}}(x)$ and conditional distribution $p(x(t_{f})\;|\;x(t_{i}))$ consistent with $N^{*}$. In such cases, equation (4.1) does not provide a stronger bound on EP than the conventional second law. This is not as much of a problem as one might fear though. For *every* unit structure $N^{*}$, there are initial distributions $p_{t_{i}}(x)$ and conditional distributions $p(x(t_{f})\;|\;x(t_{i}))$ that are consistent with $N^{*}$ where the r.h.s. of equation (4.1) is non-negative, so that the bound in equation (4.1) is at least as strong as the conventional second law. This is my third and final main result. (This result is presented in electronic supplementary material, proposition F.2, and is also proven in electronic supplementary material, appendix F, based on results in electronic supplementary material, appendix I.)

## 5. Thermodynamics of feedback control for composite systems

We can use equation (4.1) to extend previous work on the thermodynamics of feedback control [17,27,28] to account for a known set of dependency constraints of the system being controlled. Suppose we have a composite system with some associated unit structure $N^{*}$ and some desired initial and final joint distributions over the states of the system, $p_{t_{i}}^{†}(x)$ and $p_{t_{i}}^{†}(x)$, respectively. Suppose we also have a feedback controller, C, whose state space C has values c. Before the system starts to evolve, the controller observes the initial state of the system through a noisy channel, p(c|x). This observation does not affect that initial system state, i.e. there is no back-action. So the initial joint distribution immediately after the observation is

5.1 $$p_{t_{i}}(c,x)=p(c|x)p_{t_{i}}^{†}(x).$$

As is standard in the literature of the thermodynamics of feedback control, we do not consider the thermodynamics of this measurement process. Note that $p_{t_{i}}(x)=p_{t_{i}}^{†}(x)$.

After the measurement, c does not change. However, the system can observe c as it evolves. The result is a new final distribution,

5.2 $$p_{t_{f}}(c,x)=\sum_{x^{\prime }}p(c|x^{\prime })p_{t_{i}}^{†}(x^{\prime })p(x_{t_{f}}|x_{t_{i}}=x^{\prime },c),$$

where we abuse notation and write $p(x_{t_{f}}|x_{t_{i}},c)$ for the distribution over final states of the system conditioned on the initial state being $x^{\prime }$ and the feedback process state being c. For simplicity, we parallel the conventional analysis in the literature and require that the marginal final distribution obeys $\sum_{c}p_{t_{f}}(c,x)=p_{t_{f}}^{†}(x)$. In order to analyse the thermodynamics of feedback control, one must define a Hamiltonian over the states of the system, so that one can define the work on/from the system. Following convention, I assume the Hamiltonian is uniform at both $t_{i}$ and $t_{f}$, and assume it is related to the global rate matrix via LDB.

Let $N^{*}$ be some unit structure with height less than three representing the original system, without the feedback apparatus. Using equation (4.1), the EP without the feedback apparatus is lower-bounded by

5.3 $$\sigma^{N}\geq B_{N^{*}}=I^{N^{*}}(p_{t_{i}}^{†}(X))-I^{N^{*}}(p_{f}^{†}(X)).$$

By coupling that original system to the feedback apparatus we construct a new system, M, which comprises the original system together with an extra subsystem (the feedback apparatus) and new dependencies of the original coordinates of the system on the state of that new subsystem. There are many possible unit structures, $M^{*}$, over this new joint system-feedback-apparatus. For simplicity, exploit the fact that C evolves independently of the other coordinates in the system (by not evolving at all) to construct $M^{*}$ directly from $N^{*}$, by replacing each unit $\omega \in N^{*}$ with a new unit, $\omega^{\prime }(\omega ):=\omega \cup C$. So $M^{*}$ and $N^{*}$ contain the same number of units, with each unit in $M^{*}$ containing the subsystem C, and $X_{\omega^{\prime }(\omega )}=C\times X_{\omega }$. (It does not matter if we add an additional unit to $M^{*}$, containing just C itself.) This gives a new lower bound on the EP, $B_{M^{*}}$. In electronic supplementary material, appendix J, it is shown that the difference between the lower bound on EP in the new, feedback scenario and the lower bound on EP in the original, no-feedback scenario, is

5.4 $$B_{M^{*}}-B_{N^{*}}=\Delta \left[{\hat{\sum }}_{\omega \in N^{*}}I(X_{\omega };C)\right]-\Delta I(X_{N};C).$$

By conservation of energy, the work done on the system during $\left[t_{i},t_{f}\right]$ is the change in its internal energy minus the heat flow to all the reservoirs, which is given by the sum of the (temperature-normalized) entropy flows to the reservoirs. (Equivalently, this is the negative of the work extracted from the system.) Since the Hamiltonian is uniform at $t_{i}$, $t_{f}$, the change in internal energy is zero. For simplicity assume all reservoirs have the same temperature, T, and choose units so that $k_{B}T=1$. Then that sum of entropy flows is the total change in the entropy of the system minus the EP.

Combining this with equation (4.1), it is shown in electronic supplementary material, appendix J that the amount of work that can be extracted from the system under feedback control if one takes into account the unit structure is (perhaps loosely) upper-bounded by

5.5 $$\Delta \left[{\hat{\sum }}_{\omega \in N^{*}}S(X_{\omega }|C)\right].$$

By contrast, the conventional analysis in the literature, in which one does not account for the unit structure of the system, results in an upper bound of $\Delta S(X_{N}|C)$ [17,27,28]. The difference between these two terms is how much the unit structure restricts the amount of work we can extract from a system by observing its state.

## 6. Examples of the strengthened second law

In this section, I work through some elementary examples illustrating equation (4.1). All unit structures in these examples are implicitly assumed to have height less than 3.

### (a) Example 1

Consider any process where every coordinate that is in the intersection of two or more distinct units stays constant throughout. In such a process

6.1 $$-\Delta I^{N^{*}}=\left[\sum_{\omega \in \Gamma_{N^{*}}^{R}}S(p_{\omega }(t_{i}))-S(p_{\omega }(t_{f}))\right]-[S(p(t_{i}))-S(p(t_{f}))],$$

where the sum runs only over the root nodes. Moreover, since any unit that never changes its state generates no EP, in this kind of process

6.2 $${\hat{\sum }}_{\omega }\sigma^{\omega }=\sum_{\omega }\sigma^{\omega },$$

by the definition of in-ex sum. The lowest each $\sigma^{\omega }$ can be is zero (which occurs when each unit ω evolves semi-statically slowly). Therefore we can combine equation (6.2) with equations (3.21) and (6.1) to establish that the lower bound on EP is

6.3 $$B_{N^{*}}=\left[\sum_{\omega }S(p_{\omega }(t_{i}))-S(p_{\omega }(t_{f}))\right]-[S(p(t_{i}))-S(p(t_{f}))],$$

exactly, i.e. equation (6.1) is a strict lower bound on the EP. This lower bound holds no matter what $p_{t_{i}}(x)$ and $p(x(t_{f})\;|\;x(t_{i}))$ are, so long as $p(x(t_{f})\;|\;x(t_{i}))$ is consistent with the unit structure.

As an illustration of this result, suppose that no two units intersect one another, and that every unit contains just a single coordinate. Then the lower bound on EP is

6.4 $$B_{N^{*}}=\left[\sum_{i}S(p_{i}(t_{i}))-S(p_{i}(t_{f}))\right]-[S(p(t_{i}))-S(p(t_{f}))],$$

which is the drop among the coordinates in their multi-information, sometimes called ‘total correlation’. (This lower on the EP was previously derived in [17,43], in the special case that each coordinate is a physically separate subsystem.) By repeated application of the data-processing inequality, it is easy to confirm that this lower bound on the EP is non-negative.

Note though that equation (6.1) holds for *any* process with a height 2 unit structure, so long as the ending entropies of (the joint coordinates in the units corresponding to) the leaf nodes equal the associated starting entropies. In particular, this is true even if the coordinates in the leaf nodes *do* change state during the process. Since the dependency graph has height 2, equation (4.1) tells us that the expression in equation (6.1) is a lower bound on the EP of such a process. Furthermore, the same argument using the data-processing inequality establishes that that lower bound is non-negative. However, in general, if the coordinates in the leaf nodes change their states during the process, that lower bound may not be tight.

### (b) Example 2

Suppose that a system comprises three physically separated subsystems, {1,2,3}, each with two possible states, 0 and 1. Suppose as well that the dynamics can be represented with the height-2 unit structure $A^{*}=\{\{1,2\},\{2\},\{2,3\}\}$. So subsystem 2 evolves independently, while the dynamics of both subsystems 1 and 3 depend on the state of subsystem 2.

Suppose as well that initially, $x_{1}=x_{3}$ with uniform probability over their two possible joint states, and that $x_{2}$ is independent of both $x_{1}$ and $x_{3}$, also with uniform probability over its states:

6.5 $$p_{t_{i}}(x)=\frac{1}{4}\sum_{k=0}^{1}\delta (x_{1}(t_{i}),k)\delta (x_{3}(t_{i}),k)\sum_{m=0}^{1}\delta (x_{2}(t_{i}),m).$$

Therefore, $S(p(t_{i}))=2\ln2$, and so

6.6 $$\begin{array}{rl} I^{A^{*}}(p(t_{i})) & \;=[2\ln2+2\ln2-\ln2]-2\ln2 \end{array}$$

6.7 $$\begin{array}{rl} & \;=\ln2 \end{array}$$

Assume that $x_{2}$ eventually loses all information about its initial state. So

6.8 $$p(x_{2}(t_{f})\;|\;x(t_{i}))=p(x_{2}(t_{f})\;|\;x_{2}(t_{i}))=p(x_{2}(t_{f})).$$

In addition, as required by the unit structure, have $x_{1}$ and $x_{3}$ evolve independently of one another, conditioned on the state $x_{2}$, and presume that they both eventually lose all information about their own initial states and the initial state of $x_{2}$. So for example,

6.9 $$p(x_{1}(t_{f})\;|\;x_{2}(t_{f}),x_{1}(t_{i}),x_{2}(t_{i}))=p(x_{1}(t_{f})\;|\;x_{2}(t_{f})).$$

Combining,

6.10 $$\begin{array}{rl} & p(x(t_{f})\;|\;x(t_{i}))=p(x_{1}(t_{f}),x_{3}(t_{f})\;|\;x_{2}(t_{f}),x(t_{i}))\;p(x_{2}(t_{f})\;|\;x(t_{i}))\\ & \;=p(x_{1}(t_{f})\;|\;x_{2}(t_{f}),x_{1}(t_{i}),x_{2}(t_{i}))\;p(x_{3}(t_{f})\;|\;x_{2}(t_{f}),x_{2}(t_{i}),x_{3}(t_{i}))\;p(x_{2}(t_{f})) \end{array}$$

6.11 $$\begin{array}{rl} & \;=p(x_{1}(t_{f})\;|\;x_{2}(t_{f}))\;p(x_{3}(t_{f})\;|\;x_{2}(t_{f}))\;p(x_{2}(t_{f})). \end{array}$$

Therefore, $S(X(t_{f}))=S(X_{1}(t_{f})\;|\;X_{2}(t_{f}))+S(X_{3}(t_{f})\;|\;X_{2}(t_{f}))+S(X_{2}(t_{f}))$, and so

6.12 $$\begin{array}{rl} & I^{A^{*}}(p(t_{f}))=-S(X(t_{f}))\\ & \;\;+[(S(X_{1}(t_{f})\;|\;X_{2}(t_{f}))+S(X_{2}(t_{f})))+(S(X_{3}(t_{f})\;|\;X_{2}(t_{f}))+S(X_{2}(t_{f})))-S(X_{2}(t_{f}))]=0. \end{array}$$

Combining equations (6.7) and (6.12) establishes that the EP is lower-bounded by ln⁡2. Note that we can derive this lower bound on the EP even though both subsystems 1 and 3 are continually observing subsystem 2 during the process, even if subsystem 2’s state is changing as they observe it. In addition, this lower bound holds no matter what the ending distribution $p_{t_{f}}(x)$ is, so long it can be written as in equation (6.11). (So in particular, as discussed in the introduction, it applies to a simple extension of the cell-sensing scenario analysed in [21,22].)

### (c) Example 3

**Figure 2.**
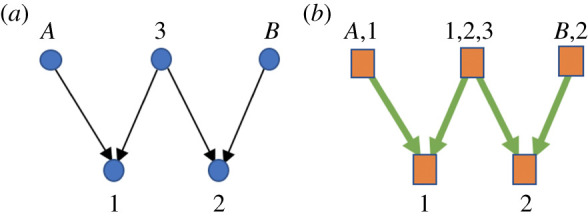
The random walker scenario described in the Introduction and investigated in example 3. (*a*) In the left panel, the five coordinates are indicated by circles, with the associated rate matrix dependencies indicated by arrows, using the same convention as in figure 1. (*b*) The right panel shows a height-2 dependency graph for this rate matrix. Each square is a different unit, with the associated coordinates explicitly written. Note that in dependency graphs arrows indicate the partial order of subset inclusion. In this example, the number of units is the same as the number of coordinates, but that need not be the case in general. (Online version in colour.)

Return to the example of a random walker presented in the Introduction, with an associated height 2 unit structure illustrated in figure 2. Plugging into equation (3.18) and using obvious shorthand,

6.13 $$\begin{array}{rl} -\Delta I^{N^{*}} & \;=\Delta [S(N)-S(1)-S(2)-S(1,2,3)-S(A,1)-S(B,2)\\ & \;\;+(S(1)+S(1)+S(1)+S(2)+S(2)+S(2))] \end{array}$$

6.14 $$\begin{array}{rl} & \;=\Delta [S(A,B\;|\;1,2,3)+S(1)+S(2)-S(A\;|\;1)-S(B\;|\;2)]. \end{array}$$

Suppose that at $t_{i}$ the full system has a single specific state with probability 1. So $S(t_{i})=0$. Suppose as well that the position in the lattice is uniformly random at $t_{f}$. (For example, this will occur at large enough $t_{f}$ if the lattice has periodic boundary conditions and both $X_{1}$ and $X_{2}$ evolve by randomly choosing one of their two neighbours.) This means that knowing the values of $x_{2},x_{3}$ at $t_{f}$ tells us nothing about the most recent values of $x_{1}$ not already given by the value of $x_{1}$ at $t_{f}$, and so in particular tells us nothing about the most likely value of $x_{A}$ then. The same is true concerning the value of $x_{B}$ at $t_{f}$. This all means that $S_{t_{f}}(A,B\;|\;1,2,3)=S_{t_{f}}(A\;|\;1)+S_{t_{f}}(B\;|\;2)$.

Combining gives $-\Delta I^{N^{*}}=2\ln L$. So equation (4.1) provides a strictly positive lower bound on the global EP. Note that this lower bound applies no matter what the dynamics of the process; it can be quasi-statically slow, it can involve Hamiltonian quenches, but so long as the unit structure does not change during the process, the EP is lower-bounded by 2ln⁡L. Furthermore, so long as the Hamiltonian is uniform at both $t_{i}$ and $t_{f}$, the total work extracted in the process is the gain in entropy of the full system minus the global EP. Combining establishes that the total work extracted is upper-bounded by

6.15 $$S_{t_{f}}(A\;|\;1)+S_{t_{f}}(B\;|\;2)+2\ln N.$$

Note that increasing N while keeping LN constant means that the precise value $\left(x_{1},x_{2}\right)$ tells us less about the precise lattice position. Equation (6.15) tells us that increasing the significance of $x_{3}$ this way increases the upper bound on the total amount of work that can be extracted.

## 7. Discussion

In this paper, I consider the thermodynamics of multi-dimensional systems evolving according to a continuous-time Markov chain. My main result is a strengthened version of the conventional second law, which applies whenever we have an *a priori* set of ‘dependency constraints’ that for each coordinate i specify which other coordinates can directly affect the dynamics of i, via the rate matrix. The result holds for any coordinate system—the coordinates can be conventional phase space coordinates, they can be states of a set of separate interacting subsystems of an overall system, they can be positions in a sequence of more refined coarse-grainings of the state of the system, they can involve amounts of various chemicals in the system, etc.

To derive my result I first translate the dependency constraints into a ‘unit structure’. This gives a sigma algebra that groups the coordinates into overlapping sets, in a way that respects the dependency constraints. In general, any set of dependency constraints can be translated into more than one unit structure. In turn, any unit structure specifies an information-theoretic functional of distributions over the states of the system, called the ‘in-ex information’. To illustrate this, suppose the dependency constraints specify that each coordinate evolves autonomously, independent of the others. (As an example, this would be the case for the spatial coordinates of a particle freely evolving under over-damped Langevin dynamics in a uniform medium with no external forces.) We could then choose a unit structure that assigns each coordinate to its own unique unit. In this case the in-ex information reduces to the total correlation (sometimes called ‘multi-information’) of the system’s distribution, with each coordinate viewed as a separate random variable.

The strengthened version of the second law derived in this paper says that the EP of the system is lowerbounded by the difference between the beginning and ending values of the system’s in-ex information. This lower bound is independent of all features of the dynamics other than the beginning distribution, the ending distribution and the dependency constraints restricting how the dynamics could have caused the initial distribution to evolve into the ending distribution. Accordingly, we can use this strengthened second law to upper-bound the amount of work that can be extracted from a system as it evolves from one specified distribution to another [27,47], in a way that accounts for dependency constraints governing the system’s dynamics. Similarly, this strengthened second law can be used to refine recent results in thermodynamics of feedback control [48], to account for dependency constraints in the system being controlled.

In contrast to other similar recently derived lower bounds on EP [19,20], the one derived here does not require that the dynamics of the system be a multipartite process. Nor does it require that local detailed balance holds. These two features mean the lower bound applies to any system undergoing continuous-time Markovian dynamics, even if the system has no natural thermodynamic interpretation. As a result, we can apply these results to everything from (Markov models of) evolving opinion networks to replicator dynamics of a population of evolving organisms.

A recent paper [17] used an information-geometric analysis to also derive bounds on minimal EP that arise due to constraints on the rate matrix of a system’s dynamics.

To use the analysis in [17] one needs to first find an operator ϕ over the set of all joint distributions which both obeys the Pythagorean theorem of information theory and which commutes with the time-evolution operators defined by the set Λ of allowed rate matrices. In general, there are many such ϕ, but different ones will result in different bounds on EP.

The analogue of Λ in this paper is the set of dependency constraints. The analogue to finding one (or more) ϕ’s for the approach in this paper is choosing a coordinate system and associated unit structure that represents the dependency constraints and is rich enough for the lower bound on EP to be strictly positive. Similarly to the case with the approach in [17], where different ϕ all consistent with the constraints on the set of allowed rate matrices will result in different bounds on EP, in general different unit structures all consistent with the constraints on the set of allowed rate matrices will result in different bounds on EP.

Kolchinsky & Wolpert [17] provide many examples of how constraints on the allowed rate matrices can be used to derive non-zero lower bounds on EP, including collective flashing ratchets, Szilard boxes where the particle is subject to a gravitational force in addition to driving by a piston, Szilard boxes where there are constraints on the way the piston can be used, evolving Ising spin systems, etc. Many of these examples can be formulated as systems evolving under dependency constraints (e.g. most of the examples involving in [17] ‘modularity constraints’ can be directly formulated this way). Future work involves comparing the EP bounds in this paper with the ones in [17], and more generally trying to synthesize the two approaches.

## Data Availability

All scripts used in this study are openly accessible through https://github.com/StochasticBiology/boolean-efflux.git. The data are provided in electronic supplementary material [49].

## Footnotes

1 It is important to note that this separation is between the timescale of the dynamics of the overall system and the timescale of the implicit dynamics of the thermodynamic reservoirs that are coupled to the system [1]. It does *not* concern the time-scales of the dynamics of the different coordinates of the system. For analysis of the latter kind of time-scale separation in the special case of a bipartite system, see [31], and for a more general analysis, see [32].

2 Note that assuming the state space of the system is a Cartesian product does not limit the applicability of the analysis. Suppose that the set of physically allowed states are a subset of such a Cartesian product, $Y\subset \prod_{i}X_{i}$, but that Y is not itself such a Cartesian product. We can model such a scenario using the Cartesian product state space $X=\prod_{i}X_{i}$, simply by restricting the rate matrix of the CTMC so that there is zero probability of going from a state in Y to a state in X∖Y.

3 One can always construct a unit structure for any CTMC, and in general there are multiple possible unit structures. Not all collections of units is a unit structure though; one can form a collection of units that contains two units $\omega ,\omega^{\prime }$, but not the unit $\omega \cap \omega^{\prime }$, and so that collection would not be a unit structure.

4 Formally, if $K_{x}^{x^{\prime }}(t)\neq 0$ and $x_{i}^{\prime }\neq x_{i}$ for all i∈α, then there is some unit $\omega \in N^{*}$ where α⊆ω.

5 For a vacuous unit ω, the dynamics of $x_{\omega }$ is fully specified by the rate matrices of the subunits $\omega^{\prime }\subseteq \omega$, and so to specify a rate matrix for ω would be redundant.

6 Physically, L(v) will often reflect an interaction Hamiltonian coupling the coordinates in L(v) without any back-action of the value of $x_{P(v)}$ onto the values of $x_{L(v)\setminus P(v)}$. This is not necessary for the analysis below though.

7 To see this, first use the fact that for every unit ω in any unit structure, whether that unit structure is tight or not, ω⊆L(ω). Then note that if ω⊂L(ω), at least one reservoir in L(ω) would violate the requirement that the unit structure be tight.

8 To see this, first note that −ω⊆P(−ω), since any set is contained in the associated puppet set. In addition, if there were coordinates i∈P(−ω) that lay inside ω for some reservoir v, then there would be a coordinate j∈−ω such that P(v) contains both i and j. That in turn would mean that j∈P(ω) and therefore j∈L(ω). However, that violates the assumption that the unit structure is tight. Therefore P(−ω)⊆−ω. Combining establishes the claim.

9 Note that for convenience in the definition of ‘in-ex information’ below, I adopt the convention that $\left\langle \dot{Q}(t)\right\rangle$ is the expected EF rate *out of* the system into the reservoirs, and so is the negative of the integrand in equation (1.3).

10 A related result was derived in [17], applicable to the special case where the composite system is an MPP, and it obeys a form of LDB. The derivation here is the first that shows the result applies more generally, even when those two conditions are violated.

11 As an example, if one of the units ω has a set of units within it, and if each of those units *within* ω has zero local EP but that set of units within ω has non-zero change in its in-ex information during the process, then the local EP of ω as a whole is non-zero.

12 It is also shown explicitly in that appendix there are unit structures of arbitrary height that obey equation (4.1), in addition to unit structures that violate it. See electronic supplementary material, proposition F.2.
